# Biogenic Photo-Catalyst TiO_2_ Nanoparticles for Remediation of Environment Pollutants

**DOI:** 10.1021/acsomega.2c01763

**Published:** 2022-07-20

**Authors:** Boya Palajonnala Narasaiah, Pravallika Banoth, Angel Guillermo Bustamante Dominguez, Badal Kumar Mandal, Challa Kiran Kumar, Crispin H. W. Barnes, Luis De Los Santos Valladares, Pratap Kollu

**Affiliations:** † CASEST, School of Physics, University of Hyderabad, Prof. C. R Rao Road, Gachibowli, Hyderabad 500046, Telangana, India; ‡ Laboratorio de Cerámicos y Nanomateriales, Facultad de Ciencias Físicas, Universidad Nacional Mayor de San Marcos, Ap. Postal 14-0149, Lima 15081, Peru; § Department of Chemistry, School of Advanced Sciences, Vellore Institute of Technology, Vellore 632014, Tamil Nadu, India; ∥ Technology Mission Division, Department of Science and Technology, Ministry of Science and Technology, MoS&T, New Delhi 110030, India; ⊥ Cavendish Laboratory, Department of Physics, University of Cambridge, J.J. Thomson Avenue, Cambridge CB3 0HE 2, U.K.; # School of Materials Science and Engineering, Northeastern University, No 11, Lane 3, Wenhua Road, Heping District, Shenyang, Liaoning 110819, People’s Republic of China; ○ School of Physics, University of Hyderabad, Prof. C. R Rao Road, Gachibowli, Hyderabad 500046, Telangana, India

## Abstract

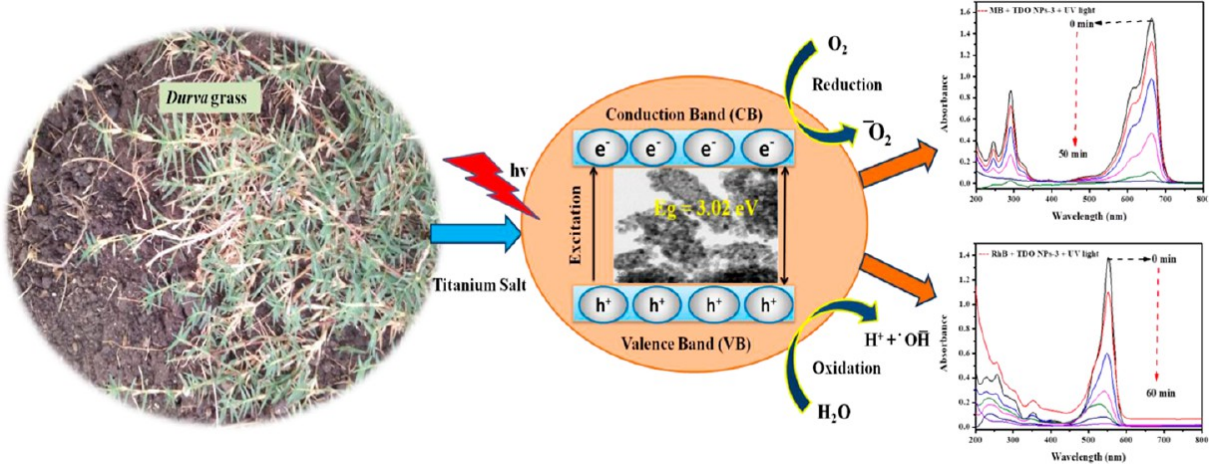

This article reports a benign environmentally friendly fabrication method of titanium dioxide (TDO) nanoparticles (named TDO NPs3, TDO NPs5, and TDO NPs8) using aqueous extract of *durva* herb waste. This synthesis process avoids use of harmful substances and persistent chemicals throughout the order and enables us to control the size of the nanomaterials. Characterization of TDO nanoparticles was analyzed by ultraviolet–visible spectroscopy, X-ray diffraction, and Fourier transform infrared spectroscopy. The morphological nature of the TDO samples was inspected by transmission electron microscopy, which indicated that the TDO NPs3, TDO NPs5, and TDO NPs8 were spherical in shape, with average sizes of 5.14, 12.54, and 29.61 nm, respectively. The stability of TDO nanoparticles was assessed using thermogravimetric analysis and dynamic light scattering analysis. These samples could be used for degradation of polluting industrial textile dyes, such as methylene blue (MB) and rhodamine B (Rh-B). Remarkably, the TDO NPs3 sample (5.14 nm size) exhibits a noticeable degradation of the MB dye in a shorter time period (50 min) than the TDO NPs8 sample with a size of 29.61 nm (120 min). The TDO NPs3 sample was also tested for degradation of Rh-B dye, showing high degradation efficiency over a short period of time (60 min). In contrast, the TDO NPs8 sample showed degradation of the Rh-B dye in 120 min. The effect of the dye concentration and the catalyst dose to remove dye pollutants has also been investigated. The synthesized TDO NPs act as exceptional catalysts for the degradation of dyes, and they are promising materials for the degradation of industrial polluting dyes.

## 1 Introduction

Nanotechnology involves manipulating matter at the nanoscale.^1^ Due to an increased surface to volume ratio, extreme physical and chemical changes occur in materials at this level. In recent years, nanotechnology has rapidly gained popularity with its applications in science and technology for creating new materials at the nanoscale level that are beneficial to various areas such as the economy and the environment.^2^ Recent studies have featured potential applications of TiO_2_ nanoparticles (NPs) in various fields such as pigments, photo-catalysis, solar energy conversion, sensor technology, and biological activity such as antibacterial activities, anticancer activity, and antifungal activities.^3,4^ In spite of their wide range of applications, TiO_2_ NPs are characterized by a number of miscellaneous properties, such as high chemical stability, high redox potential, low cost, and eco-friendly nature.^5,6^ In addition, these properties are also heavily affected by the method by which NPs are synthesized, such as the specific surface area, crystal structure, particles shape, and crystallite size.^7^ TiO_2_ NPs have three distinct polymorph crystalline forms, that is, the tetragonal structure (rutile), orthorhombic structure (brookite), and tetragonal structure (anatase). Rutile is the stable phase and can be synthesized by heating at high temperatures from anatase and brookite.^8^ Each crystalline phase exhibits different physical properties depending on its structure and particle size, making it suitable for a range of applications. For example, a band gap of 3.2 eV indicates that the anatase phase can be activated under exposure to ultraviolet light, and thus, it is often used in photo-catalysis.^9^

There is an increasing demand for biological methods, which use plant components and microbes, for the synthesis of nanoscale materials since they avoid high pressure and temperature which takes more time and is not friendly to the environment.^10^ These can be overcome by developing an eco-friendly method to synthesize nanomaterials. In this way, some attempts have been reported such as Ag/TiO_2_ nanocomposites synthesized from *Euphorbia heterophylla* leaf extract^11^ and TiO_2_ NPs produced from *Trigonella foenum-graecum* extract^12^ and from micro-organism *Caenorhabditis elegans* Bristol strain.^13^

As coloring agents, about 10,000 commercially available dyes are used in industries including textiles, plastics, rubber, leather, cosmetics, etc., producing enormous quantities of colored wastewater. About 2% dye effluent, especially those from the textile sector, is derived from the production of over 7 × 10^7^ dyestuffs every year.^14^ Dye wastewater discharges into natural streams and rivers, causing severe environmental and aesthetic damage. This results in a serious threat to water quality and human health.^15^ The direct discharge of textile effluents containing dye concentrations higher than 1 mg/L can cause serious problems for the ecosystem due to the contamination of water and soil. Besides, most dyes contain synthetic organic compounds and complex organic structures, making them non-biodegradable, carcinogenic, and irritants to the skin.^16^

Nowadays, photocatalytic pollutant dye degradation is drawing more and more attention in the field of photodegradation methods. Thus, remediation methods using mineral acids and H_2_O are needed for a cheaper, non-toxic alternative.^17^ In this way, several methods are being used to degrade dyes, including Fenton oxidation,^18^ reverse osmosis,^19^ or photodegradation by using nanomaterials.^20^ Among all these methods, photodegradation with a multi-lamp photoreactor technique is a simple method, that is, a closed instrument setup, an easy handling process, and the reaction can be carried out at room temperature.

Therefore, the objective of the present study was to synthesize for the first time, environmentally benign TDO NP samples using agro-waste durva grass extract in the absence of chemical reducing agents, toxic solvents, or stabilizing agents. The synthesized TDO NP samples were characterized by several advanced analytical techniques such as X-ray diffraction (XRD), UV–vis spectroscopy, high-resolution transmission electron microscopy (HR-TEM), Fourier transform infrared spectroscopy (FT-IR), thermogravimetric analysis (TGA), dynamic light scattering (DLS), and BET analysis. The synthesized TDO NP sample exhibited outstanding photodegradation efficiency for the photodegradation of environmental polluting dyes such as rhodamine B (Rh-B) and methylene blue (MB) under ultraviolet light exposure.

## 2 Experimental Section

### 2.1 Materials and Methods

The chemicals of analytical grade (A.R) were purchased from Sigma-Aldrich, India, and prepared as needed. The chemicals used were titanium tetraisopropoxide (TIP) (purity 97%), Rh-B (purity 99%), MB (purity 99%), and double-distilled water (DDW), which is used as a reaction medium.

### 2.2 Preparation of Durva Grass Aqueous Extract

Approximately 3 g of dried *durva* grass powder was dispersed in 100 mL of double-distilled water and incubated for 2 h at 70 °C until a deep-green solution was formed. The grass was collated from sunflower agriculture land near the Tungabhadra River, Andhra Pradesh, India. Using a Whatman filter paper centrifugation at 5000 rpm, the aqueous extract was filtered, and the supernatant liquid was stored at 4 °C for use in synthesizing TDO NPs.

### 2.3 Synthesis of TiO_2_ NPs by Using Durva Grass Aqueous Extract

The TIP precursor of 0.05 M was dissolved in 30 mL of double-distilled water, and then, 30 mL of *durva* grass aqueous extracts of 1:1 (v/v) each was added at a rate of 1:1 (v/v) with vigorous stirring for 3 h at 80 °C until a brownish-yellow-colored precipitation was observed. After centrifuging for 30 min, the solution was collected and washed with double-distilled water to remove any residual contaminants. The precipitate was dried in an oven and ground to get a fine powder using a mortar–pestle and then annealed at three different temperatures 300, 500, and 800 °C for 3 h, and the obtained samples were labeled TDO NPs3, TDO NPs5, and TDO NPs8 samples, respectively. The dried powder samples were processed and stored for subsequent photocatalytic degradation of Rh-B and MB after being annealed.

### 2.4 Characterization of the Synthesized TDO NPs

UV–visible spectroscopy was used to characterize the synthesized TDO NPs (Jasco V-670 UV–visible double-beam spectrophotometer). The wavelength range of 200–800 nm was used for recording the absorption spectra. Based on the Fourier transform infrared (FT-IR) study, the functional group was predicted in the surface agro-waste durva grass extract and TDO NPs. For this purpose, KBr pellets were used as controls, and the dried TDO NPs were analyzed in a Shimadzu’s IR AFFINITY-1 system (JASCO’s FT-IR 4100 in the diffuse reflectance mode) at a resolution of 4 cm.

Furthermore, X-ray analysis was performed on a Bruker D8 Advance diffractometer under Cu Kα radiation (λ = 1.54°) at a scanning rate of 4°/min and a step size of 0.02°. The diffractogram was recorded from 10 to 90°. The transmission electron microscopy (TEM) analysis was carried out using a JEOL-JEM 2100 transmission electron microscope at 200 kV to determine the size and shape of NPs. Selected-area electron diffraction (SAED) patterns and crystalline plane fringe spacing were obtained by using an HR-TEM system. Moreover, the polydispersity indexes (PDIs) were measured on a Horiba scientific NP analyzer (SZ-100), and a DLS study was conducted to measure the zeta potential value of TDO NP dispersion. A scattering angle of 173° was used with the sample being interpreted at 20 °C. In addition, a suspension of the sample was measured in cell culture medium at 20 °C and 150 V. A thermogravimetric analyzer (model JSM 6390 LV, JOEL 2100F, USA) with an acceleration voltage of 100 kV was used to measure the thermal stability of the TDO NPs.

### 2.5 Photocatalytic Activity of TDO NPs

In an aqueous solution, the photocatalytic activity of the synthesized TDO NPs was tested for degradation of model pollutant MB and Rh-B dyes. The photodegradation was performed in a low-pressure Heber multilamp photoreactor with a UV lamp (125 W, λ_max_ 254 nm). In the absence of the photocatalyst, 60 mL of dye solution (10 mg/L) was placed in a 100 mL quartz tube and constantly agitated in dark (no light). UV–visible spectroscopy was used to track the dye solution’s color intensity change. The same experiment was then repeated in the photoreactor with various dosages of photocatalyst TDO NPs (10, 20, and 30 mg) in dark and under UV illumination. By measuring absorbance at regular time intervals between 200 and 800 nm, the photocatalytic degradation of the organic dyes MB and Rh-B was detected. Furthermore, the effect of dye doses (5 and 15 mg/L) on photodegradation of MB and Rh-B dyes was studied under the same conditions with a constant TDO NP photocatalyst dose. UV–visible spectroscopy was used to monitor photodegradation by measuring the dye solution’s absorbance.

## 3 Results and Discussion

Crystalline titanium dioxide nanoparticles (TDO NP) samples (TDO NPs3, TDO NPs5, and TDO NPs8) were synthesized using durva grass aqueous extract, and this acted as both reducing and size controlling agents, preventing particle agglomeration and nucleation of NPs, that is, regulated growth of NPs. As a result, we obtained TDO NP samples for studying the photocatalytic activity of dye degradation.

### 3.1 UV–Visible Analysis

**Figure 1:**
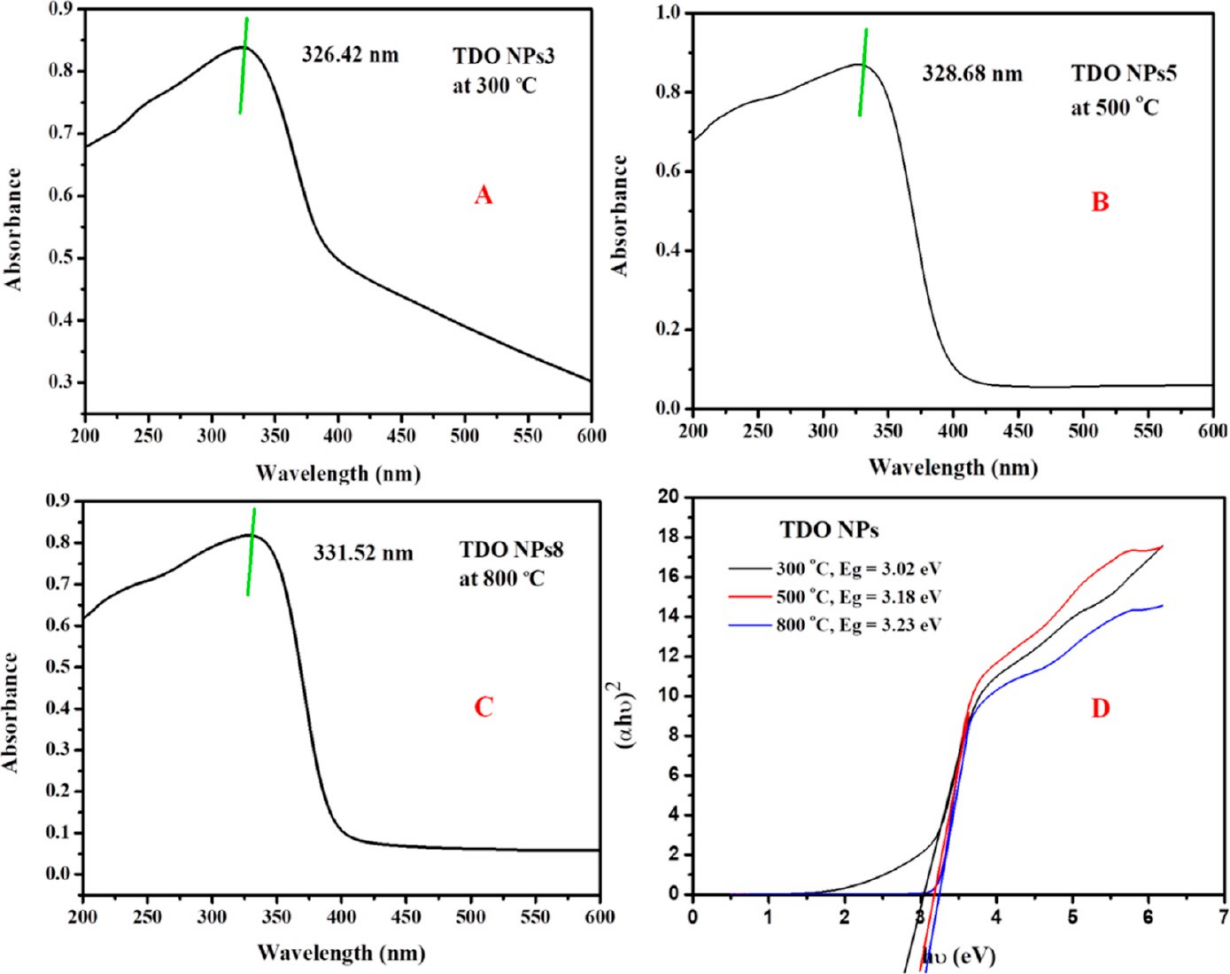
UV–visible absorption spectra of TDO NPs after annealing (A) sample at 300 °C, (B) sample at 500 °C, and (C) sample at 800 °C and (D) band gap energy of TDO NPs from 300 to 800 °C.

Figure 1 displays the UV–visible absorption spectrum of TDO NP samples. The TDO NPs3 sample had an absorption band at a maximum wavelength of 326.42 nm, which is represented in Figure 1A. TDO NPs5 had an absorption band at 328.68 nm, which is demonstrated in Figure 1B, and TDO NPs8 had an absorption band at 331.52 nm, which is noted in Figure 1C. The TDO NPs annealed at 300 °C show maximum absorption peaks in the UV range while those annealed at high temperatures between 500 and 800 °C exhibit absorption peaks in the visible range.

Therefore, from the results obtained, we concluded that the absorption properties of the TDO NPs strongly depend on their size; that is, the sample annealed at 800 °C possessing the larger-size particle distribution exhibited the absorption band in the visible range. The absorption coefficient of TDO NPs is obtained using eq 1.
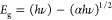
1where α is the absorption coefficient, *h*ν is the photon energy, and *E*_g_ is the optical band gap energy. The band gap energy (*E*_g_) of the TDO NP samples annealed at 300–800 °C is shown in Figure 1D. By using the Tauc plot, we have calculated the band gap energy by plotting (α*h*ν)^1/2^ on the *y* axis and the photon energy (*h*ν) on the *x* axis. The optical band gap energy of the TDO NPs annealed at 300, 500, and 800 °C are 3.02, 3.18, and 3.23 eV, respectively. Therefore, on increasing the temperature from 300 to 800 °C, the band gap energy of the TDO NPs tremendously changes, and this change might be attributed to the crystal defects in the materials. The current results are strongly analogous to reported results.^21^ Earlier studies on TiO_2_ NP-mediated *aloe vera* plant extracts estimated band gap energy to be 3.62 eV.^22^

### 3.2 Powder XRD Analysis

**Figure 2:**
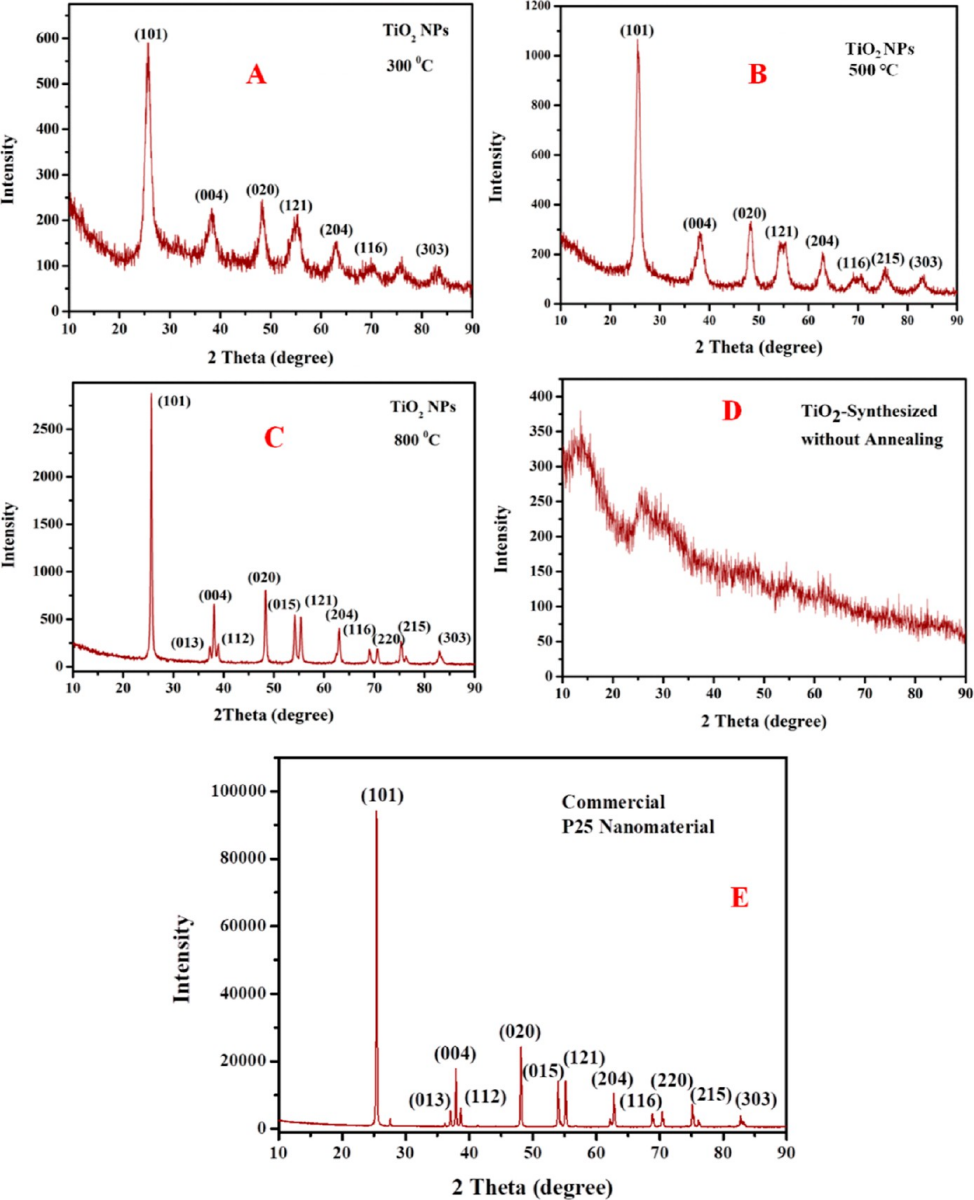
XRD pattern of TDO NPs annealed at 300 °C (A), 500 °C (B), and 800 °C (C), without annealing (D), and commercial P25 NPs (E).

XRD patterns were used to determine the structural properties of the TDO NPs to identify the phase formation and crystalline nature of the nanomaterials. Figure 2A shows the powder XRD pattern of the TDO NPs3 sample representing the characteristic diffraction peaks (*hkl* values) of the lattice planes (Miller indices) values, which are 25.56 (101), 38.42 (004), 48.41 (020), 55.03 (121), 62.94 (204), 70.37 (116), and 82.86 (303). Therefore, the diffraction pattern of the synthesized TDO NPs3 sample reveals a tetragonal structure, matching with that of standard Match3 software (JCPDS card no.: 96-500-0224). Figure 2B displays the XRD pattern of sample TDO NPs5 that shows 2θ reflection values and characteristic diffraction *hkl* values of lattice planes to be 25.79 (101), 38.52 (004), 48.44 (020), 55.22 (121), 62.96 (204), 70.42 (116), 75.71 (215), and 82.97 (303). Therefore, the TDO NPs5 sample diffraction pattern confirmed the tetragonal structure and which matches with that of standard Match3 software (JCPDS card no.: 96-500-0224). Figure 2C describes the XRD pattern of the TDO NPs8 sample that shows the 2θ reflection values and characteristic diffraction *hkl* values of lattice planes (Miller indices) to be 25.64 (101), 37.27 (013), 38.24 (004), 39.04 (112), 48.42 (020), 54.06 (015), 55.52 (121), 63.16 (204), 69.24 (116), 70.72 (220), 75.39 (215), and 83.05 (303). Therefore, the fabricated TDO NPs8 sample has a tetragonal structure, which matches with that of standard Match3 software (JCPDS card no.: 96-900-8214). However, there are no impurity peaks in XRD pattern, so we concluded that the synthesized TDO NPs8 sample was pure and crystalline in nature. However, the XRD pattern of TDO NPs synthesized without annealing suggests no crystalline phase formation, which is shown in Figure 2D. Therefore, on increasing annealing temperature from 300 to 800 °C, the crystalline nature of TDO NPs increases due to systematic arrangement of atoms in the crystal system. Figure 2E represents the XRD pattern of standard commercial P25 nanomaterial that shows 2θ reflection values and characteristic diffraction *hkl* values of lattice planes to be 25.42 (101), 37.07 (013), 37.93 (004), 38.65 (112), 48.02 (020), 53.99 (015), 55.22 (121), 62.72 (204), 68.89 (116), 70.25 (220), 75.23 (215), and 82.85 (303).

The average crystallite size was calculated using Scherrer’s eq 2
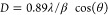
2where λ is the wavelength of X-ray radiation (1.5406 Å), β is full-width at half-maximum (fwhm) of reflection planes, and θ is Bragg’s angle. By using the above equation, we have calculated the average crystallite size and inter-planar distance (*d*) of the sample TDO NPs (Table 1). Consequently, all the samples crystallized with a tetragonal structure after annealing them from 300 to 800 °C. The average crystallite sizes obtained for these samples TDO NPs3, TDO NPs5, and TDO NPs8 are 5.88, 7.83, and 25.96 nm, respectively (Table 1). The average crystallite size of the commercial P25 nanomaterial was calculated from Scherrer’s formula, and it was around 64.84 nm. Aravind et al. (2021) synthesized TiO_2_ NPs with the average crystallite size of 31–42 nm using jasmine flower aqueous extract.^23^

**Table 1:** Structure and Geometric Parameters of TDO NPs by XRD Analysis

tem. (°C)	2θ	fwhm value	plane	*d*-spacing (Å)	cos(θ)	crystalline size (nm)
300	25.69	1.29	(101)	3.48	0.97497	6.60
	38.32	1.68	(004)	2.34	0.94460	5.22
	48.41	1.17	(200)	1.87	0.91208	7.77
	55.37	2.38	(211)	1.67	0.88551	3.94
average size						**5.88**
500	25.66	0.99	(101)	3.85	0.97503	8.60
	38.29	1.42	(004)	2.61	0.94469	6.18
	48.31	1.09	(200)	2.08	0.91244	8.35
	55.38	1.14	(211)	1.83	0.88547	8.22
average size						**7.83**
800	25.60	0.30	(101)	3.94	0.97514	28.38
	37.99	0.33	(004)	2.65	0.94554	26.60
	48.36	0.35	(200)	2.08	0.91226	26.00
	54.16	0.37	(105)	1.87	0.89037	25.20
	55.41	0.38	(211)	1.82	0.88535	24.66
average size						**25.96**

### 3.3 FT-IR Analysis

**Figure 3:**
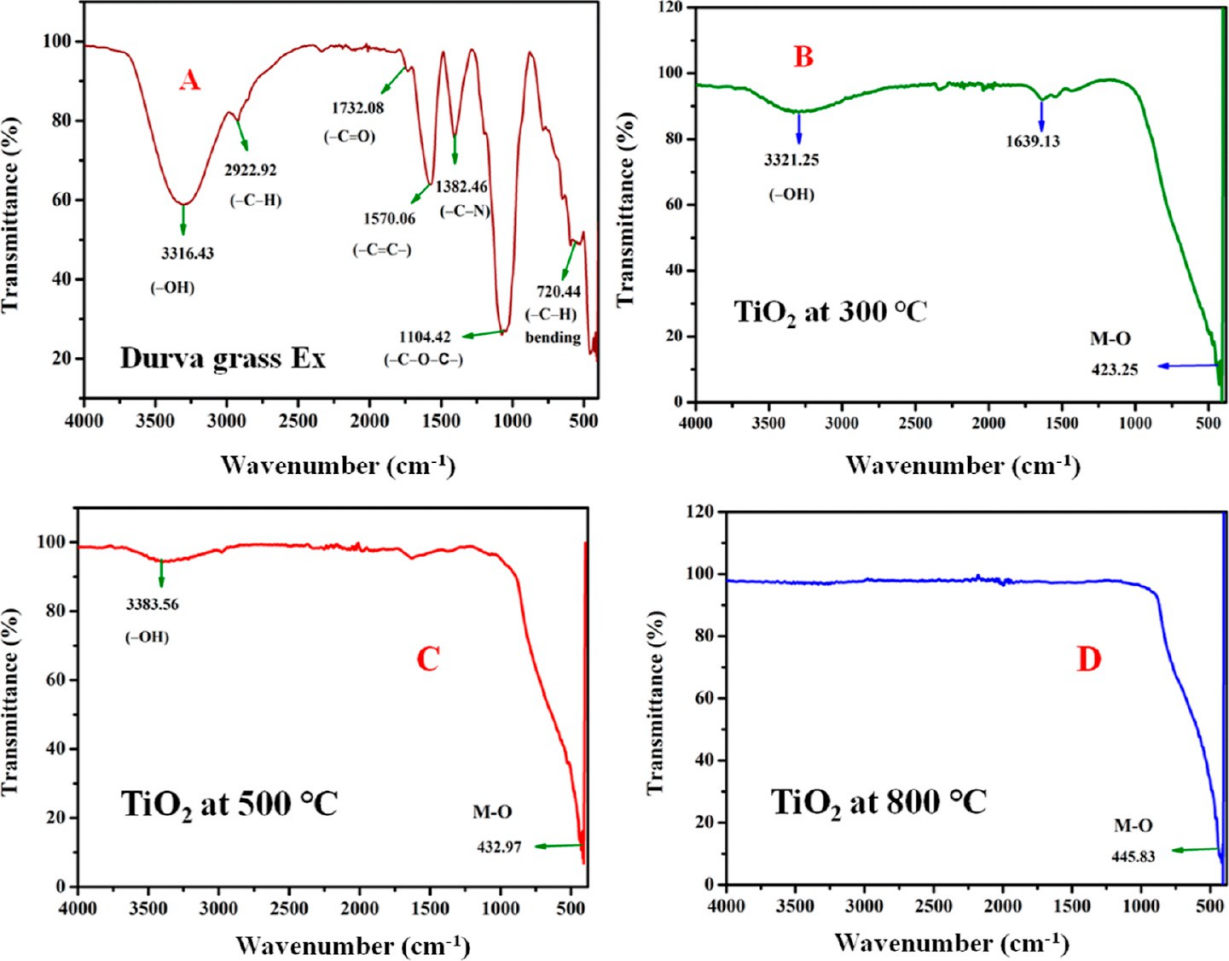
FT-IR spectra of (A) durva grass extract (B) TDO NPs3 annealed at 300 °C, (C) TDO NPs5 annealed at 500 °C, and (D) TDO NPs8 annealed at 800 °C.

The functional groups in durva grass extract and their biomolecule capping on the surfaces of TDO NPs were determined using FT-IR analysis. The FT-IR spectrum of agro-waste durva grass extract in the 500–4000 cm^–1^ range is shown in Figure 3A. As a result, the absorption characteristic peak at 3316.43 cm^–1^ is associated with the hydroxyl group (−OH) stretching vibration, indicating the presence of polyphenols. Aromatic −C–H stretching vibration could be assigned to the band at around 2922.92 cm^–1^. Carbonyl (−C=O) stretching vibration is responsible for the peak at 1732.08 cm^–1^. The peak at 1382.46 cm^–1^ could be attributed to the carbon and nitrogen (−C–N) stretching vibration of amide.

According to the spectrum, the absorption band at 1104.42 cm^–1^ corresponds to the stretching vibration of carbon, oxygen, and carbon (−C–O–C−). The flexural vibration of aliphatic carbon and hydrogen is responsible for the peak at 720.44 cm^–1^ (−C–H). Al-hamoud et al. (2022) reported phytomolecule functional groups present (3425, 2917, 2849, 1630, 1070, and 625 cm^–1^, respectively) in the *Pulicaria undulata* plant extract.^24^ The FT-IR spectrum of TDO NPs3 at 300 °C is shown in Figure 3B, with the absorption band at 3321.25 cm^–1^ corresponding to the stretching vibration of the −OH group, the peak at 1639.13 cm^–1^ corresponding to the stretching vibration of alkene (−C=C−), and the band at 423.25 cm^–1^ corresponding to the stretching vibration of titanium metal and oxygen (Ti–O) bonds.

Our current findings are consistent with those of FT-IR bands (34246, 3430, 1642, 1529, 1450, 1134, 1168, 1092, and 469 cm^–1^) of TiO_2_-supported biochar reported by Abodif et al. (2020).^25^ The FT-IR spectrum of TDO NPs5 at 500 °C is shown in Figure 3C, with the band at 3383.56 cm^–1^ being attributed to the stretching vibration of the −OH group, indicating that the NPs are covered with polyphenols. The stretching vibration of titanium metal and oxygen could be assigned to the band at 432.97 cm^–1^ (Ti–O bond). The FT-IR spectrum of TDO NPs8 at 800 °C is depicted in Figure 3D; however, the band at 445.83 cm^–1^ can also be attributed to the stretching vibration of titanium metal and oxygen (Ti–O) bond. According to the findings, as the annealing temperature increased from 300 to 800 °C, the stretching vibration of titanium metal and oxygen bond (Ti–O), the band, and the atomic diffusion increased, resulting in an increase in nanomaterials’ size.

### 3.4 TEM Analysis

**Figure 4:**
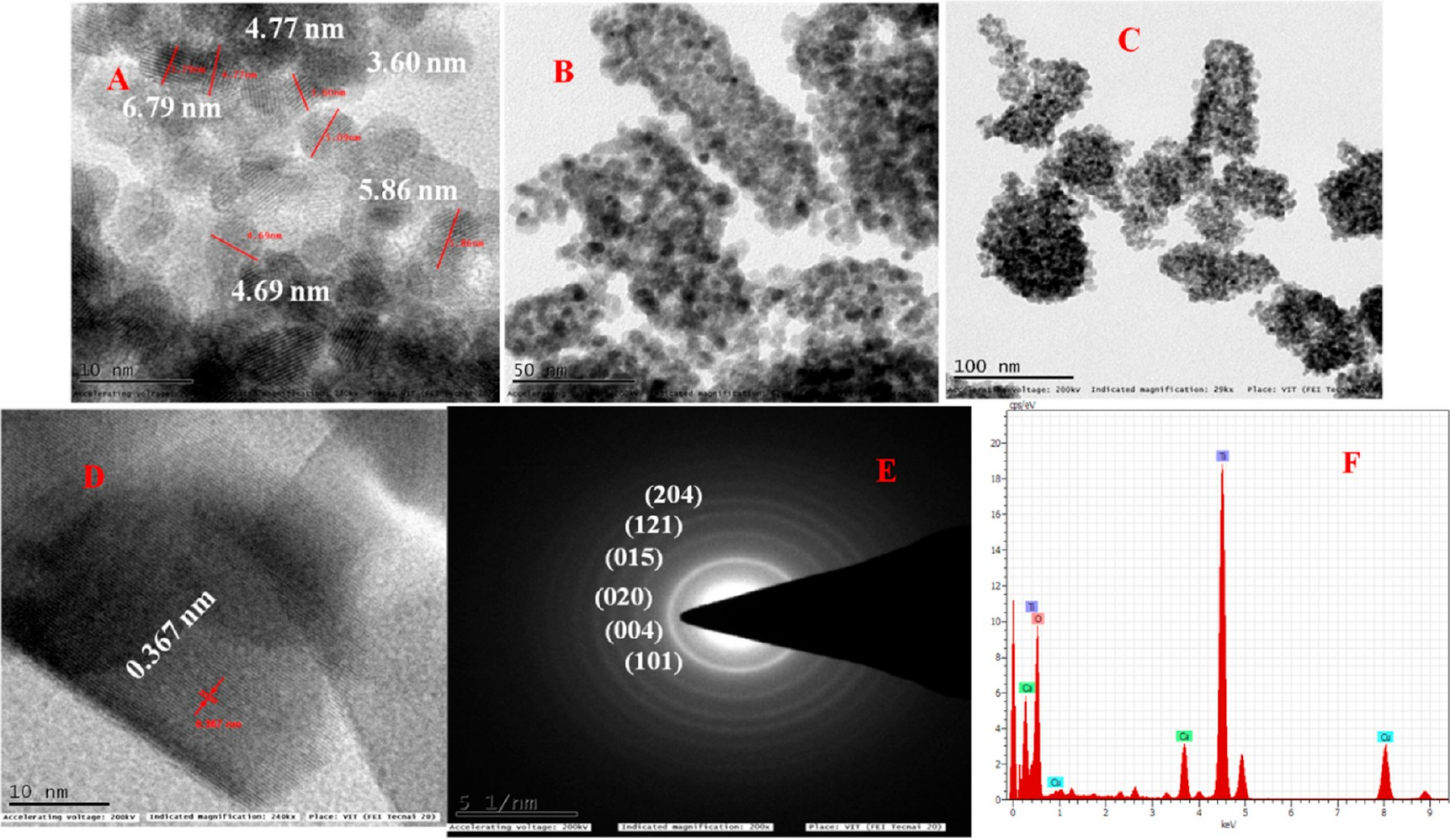
HR-TEM images of the TDO NPs3 sample annealed at 300 °C: (A) 10 nm magnification (B) 50 nm magnification, and (C) 100 nm magnification, (D) interplanar distance d values of the lattice, and (E) SAED pattern of TDO NPs and (F) existing elements in the synthesized TDO NPs.

The TEM results investigate the size, crystalline/amorphous nature, inter-planar distances, and elemental composition of nanomaterials. Figure 4 shows the HR-TEM, SAED pattern, the particle size distribution, and the EDX spectrum of TiO_2_ NPs calcined at 300 °C. The size of TDO NPs3 is in the range of 3-6 nm with a mean diameter of 5.14 nm, and the particle magnification size distribution is shown in Figure 4A–C, and the distance between the brush spacing, that is, *d*-spacing, is 0.367 nm, which characterizes the reflection plane (101) (Figure 4C). The diffuse rings in the SAED pattern of the TDO NPs3 are shown in Figure 4E, where all the bright circular rings are arranged in the SAED pattern, suggesting amorphous nature of the TDO NPs3 sample. The elemental composition of the TDO NPs3 sample is examined from the EDX spectrum.

**Figure 5:**
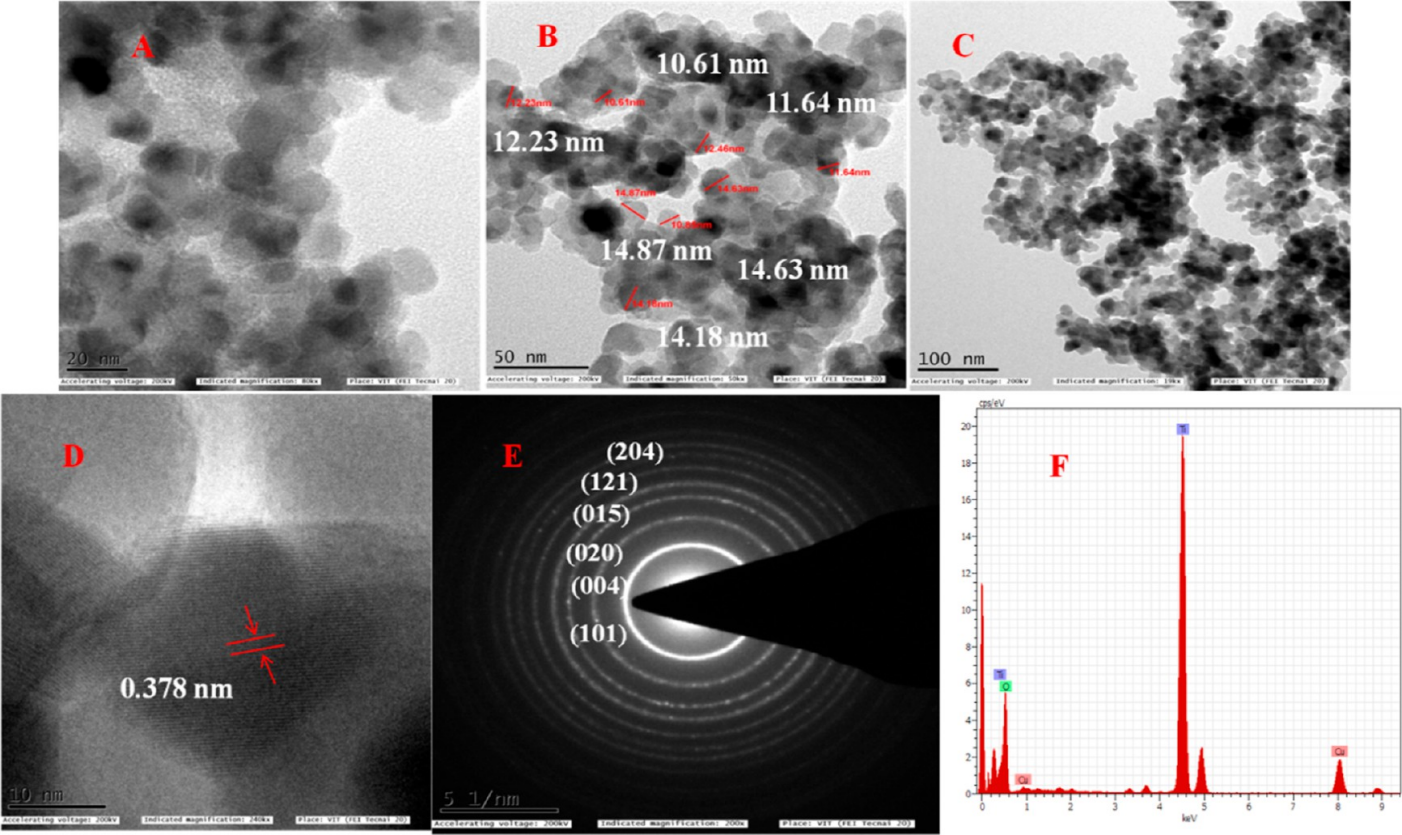
HR-TEM images of the TDO NPs5 sample annealed at 500 °C under (A) 20 nm magnification, (B) 50 nm magnification, and (C) 100 nm magnification, (D) interplanar distance d value of the lattice, (E) SAED pattern of TDO NPs, and (F) existing elements in the synthesized TDO NPs.

Figure 5A–F shows a representative image of HRTEM of TiO_2_ NPs at 500 °C with spherical particles having a mean diameter of 12.54 nm. Our results support the findings of Pushpamalini et al. (2021), where they reported that the average particle size of TiO_2_ NPs is 6.7–8.3 nm.^26^Figure 5A–C shows the particle size distribution under various magnifications of the TDO NPs5 sample with the size in the range of 8-16 nm coinciding with the XRD results according to the grid spacing plane (101). Figure 5E shows the SAED pattern of the TDO NPs5 sample; however, all bright spots in the form of concentric circular rings correspond to the polycrystalline nature of the TDO NPs5 sample. Figure 5F represents the elemental composition of the TDO NPs5 sample analyzed by EDX analysis. Therefore, the EDX spectrum shows the wt and at. % of the elements present in the sample of TDO NPs5, 62.20 wt % and 37.57 at. % of “Ti” and 22.91 wt % and 47.80 at. % of “O” atoms, and therefore, the previous results conclude that the nanomaterials obtained are pure and polycrystalline.

**Figure 6:**
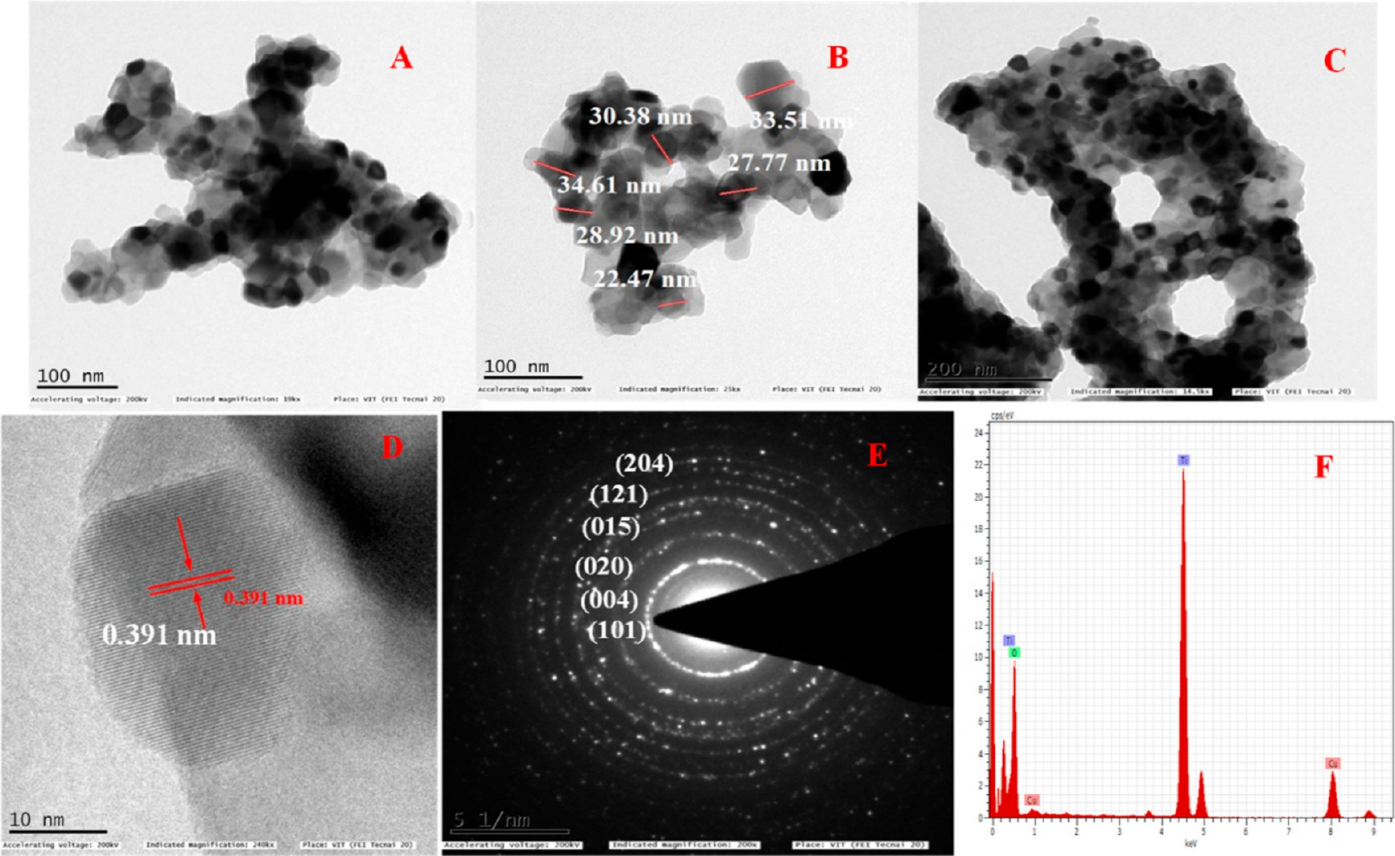
HR-TEM images of the TDO NPs8 sample annealed at 800 °C (A) under (B) 100 nm magnification (C) and 200 nm magnification, (D) interplanar distance d value of the lattice, (E) SAED pattern of TDO NPs, and (F) existing elements in the synthesized TDO NPs.

Figure 6A–F demonstrates the HR-TEM image, particle size distribution, SAED pattern, and EDX analysis of TiO_2_ NPs being annealing at 800 °C. Figure 6B shows that the particle size distribution of the TDO NPs8 sample in the range of 22–34 nm with a mean diameter is 29.61 nm. Figure 6C shows that the inter-planar distance, that is, fringe “*d*” spacing, is 0.391 nm, which strongly matches with XRD results, corresponding to the lattice spacing (101) plane. Figure 6E shows the bright spots on the circular rings in the SAED pattern, attributed to the poly-crystalline nature of the TDO NPs8 sample. Figure 6F shows the elemental composition of the sample TDO NPs8, which is obtained from EDX analysis. Therefore, elemental composition of Ti and O in wt and at. % is 63.21 and 43.84% and 22.86 and 48.23%, respectively, which endorses the purity of the NPs with polycrystalline nature of the TDO NPs8 sample.

### 3.5 DLS Analysis

**Figure 7:**
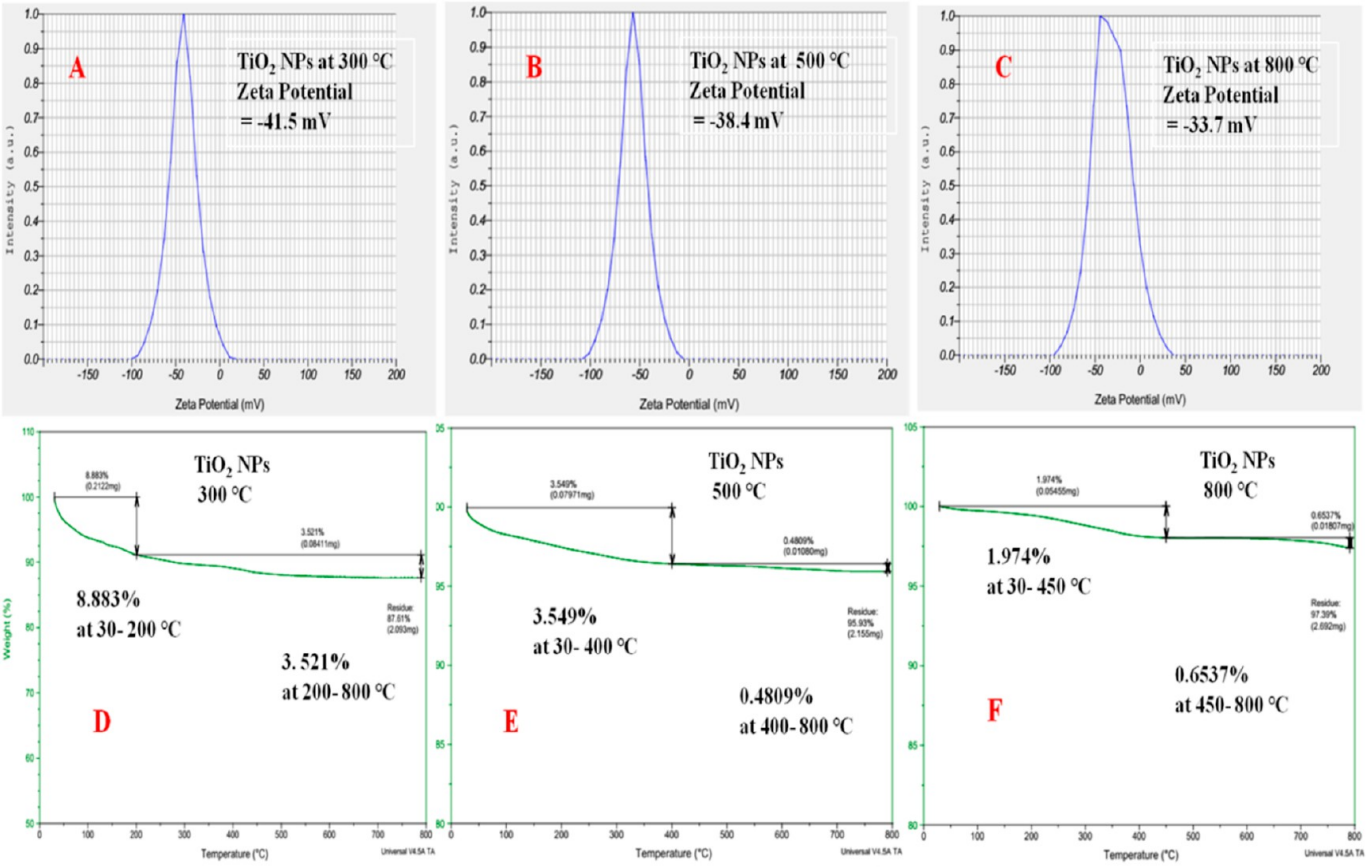
DLS spectrum of the TDO NP sample annealed at 300 °C (A), annealed at 500 °C (B), and annealed at 800 °C (C) and TGA spectrum of TDO NPs after annealing at 300 °C (D), annealing at 500 °C (E), and annealing at 800 °C (F).

Zeta potential is a key indicator for the stability of the colloidal dispersion of nanomaterials. Therefore, stability of TDO NPs was determined by the DLS method that is based on the interaction between adjacent particles or particles with similar size or mobility in colloidal dispersion of TDO NPs. From DLS analysis, the zeta potential of the TDO NPs3 sample is −41.5 mV (Figure 7A). The zeta potential of the TDO NPs5 sample is −38.4 mV (Figure 7B) and zeta potential of sample TDO NPs8 is −33.7 mV (Figure 7C). Therefore, from the results, it is clear that the higher negative values indicate very good stability of nanomaterials.^27^ From the results, we conclude that the increase in temperature from 300 to 800 °C decreases zeta potential values; hence, the stability of NPs decreases due to the smaller size (5 nm) of the NPs that show higher stability. In addition, smaller-size NPs show higher mobility compared to larger NPs. Also, the stability of the TDO NPs3 sample is the highest because it was annealed at 300 °C, but in the case of the sample being annealed at 800 °C, the sedimentation tendency of its dispersion is high, leading to lower stability compared to that of the TDO NPs3 sample.

### 3.6 Thermal Stability Analysis of TDO NPs

Thermal stability of the fabricated TDO NPs3, TDO NPs5, and TDO NPs8 samples was studied by TGA. Figure 7D shows the TGA plot of the TDO NPs3 sample, which was annealed at 300 °C. It exhibits an initial weight loss of 8.83% due to removal of the adsorbed moisture during heating between 30 and 200 °C, and a further weight loss of 3.521% was observed after heating up to 800 °C, which may be attributed to the removal of volatile compounds. Figure 7E shows the TGA plot of the TDO NPs5 sample, which was annealed at 500 °C. It exhibits an initial weight loss of 3.549% due to removal of the adsorbed moisture during heating between 30 and 400 °C, and further weight loss of 0.4809% was observed from 400 to 800 °C, which may be attributed to the removal of volatile compounds in the sample. Figure 7F shows the TGA plot of the TDO NPs8 sample, which was annealed at 800 °C. It exhibits an initial weight loss of 1.974% due to removal of the adsorbed moisture during heating between 30 and 450 °C, and further weight loss of 0.6537% was observed from 450 to 800 °C, which is attributed to the removal of volatile compounds. From these results, we conclude that among the three samples, the synthesized TDO NPs8 sample obtained after annealing at 800 °C shows very less weight loss, suggesting that this sample is pure and has a better crystallization. This is quite similar to the results obtained by Gautam et al. (2016), who reported a weight loss of 38% after heating their synthesized TiO_2_ NP sample.^28^ Therefore, the synthesized TDO NPs8 sample has high thermal stability.

### 3.7 Photoluminescence Analysis of TDO NPs

**Figure 8:**
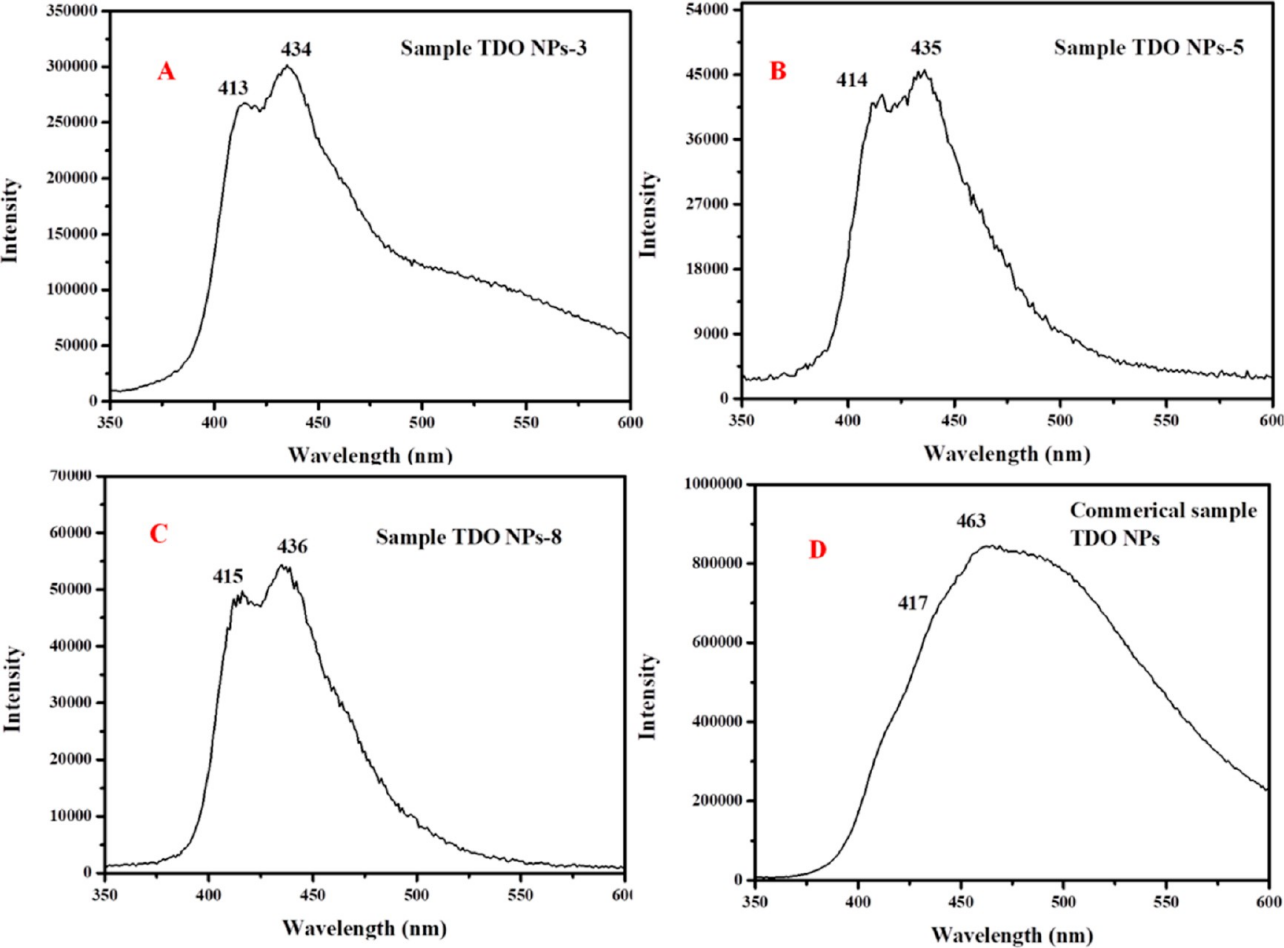
Photoluminescence spectra of TDO NPs3 (A), TDO NPs5 (B), TDO NPs8 samples (C) and commercial P25 NPs (D).

The photoluminescence (PL) studies of TDO NPs3, TDO NPs5, TDO NPs8, and standard P25 NP samples were carried out with photon-induced charge carriers in TDO NPs as shown in Figure 8. Figure 8 shows the PL spectrum of TDO NPs3, TDO NPs5, TDO NPs8 and commercial P25 nanomaterial samples. Spectra of all TDO NP samples and standard P25 nanomaterial sample were recorded at room temperature with excitation wavelength (326, 328, 331, and 328 nm), respectively. The emission photoluminescence spectrum (350–600 nm) was obtained at particular wavelengths (326, 328, 331, and 328 nm) and possessed maximum-emission bands. Figure 8A represents the emission bands of sample TDO NPs3 at 413 nm (3.00 eV) and 434 nm (2.85 eV). Figure 8B shows the PL spectrum sample TDO NPs5, which exhibits emission bands at 414 nm (2.99 eV) and 435 nm (2.85 eV). Figure 8C displays the PL spectrum sample of TDO NPs8, which exhibits emission bands at 415 nm (2.98 eV) and 436 nm (2.84). Figure 8D represents the standard P25 NP sample PL spectrum, which reveals the emission bands at 417 nm (2.97 eV) and 463 nm (2.67 eV). From the results, the emission energies of the TDO NPs are revealed to be either within the band gap or above the band gap energy (*E*_g_) of TDO NP samples that is TDO NPs3 (3.02 eV), TDO NPs5 (3.18 eV), and TDO NPs8 (3.23 eV). The emission energies of TDO NPs higher than the band gap of TDO NPs3, TDO NPs5, and TDO NPs8 samples are attributed to the recombination of electrons in the Ti^4+^ conduction band with holes in O^2–^ in the valence band. However, lower emission energies of TDO NPs than the actual band gap of TDO NPs may be due to the crystal defects or oxygen vacancies or energy levels of Ti (titanium) interstitials within the crystal system. From the results, we concluded that most of the emission energies of the TDO NPs are within the band gap of the samples.

## 4 Photocatalytic Activity of the Synthesized TDO NPs

TDO nanomaterials are used extensively in the photodegradation of industrial pollutant dyes.^29^ In the present work, MB and Rh-B dyes are chosen for the photodegradation of industrial pollutant dyes under UV irradiation at room temperature.

### 4.1 PhotoDegradation of MB Dye

**Figure 9:**
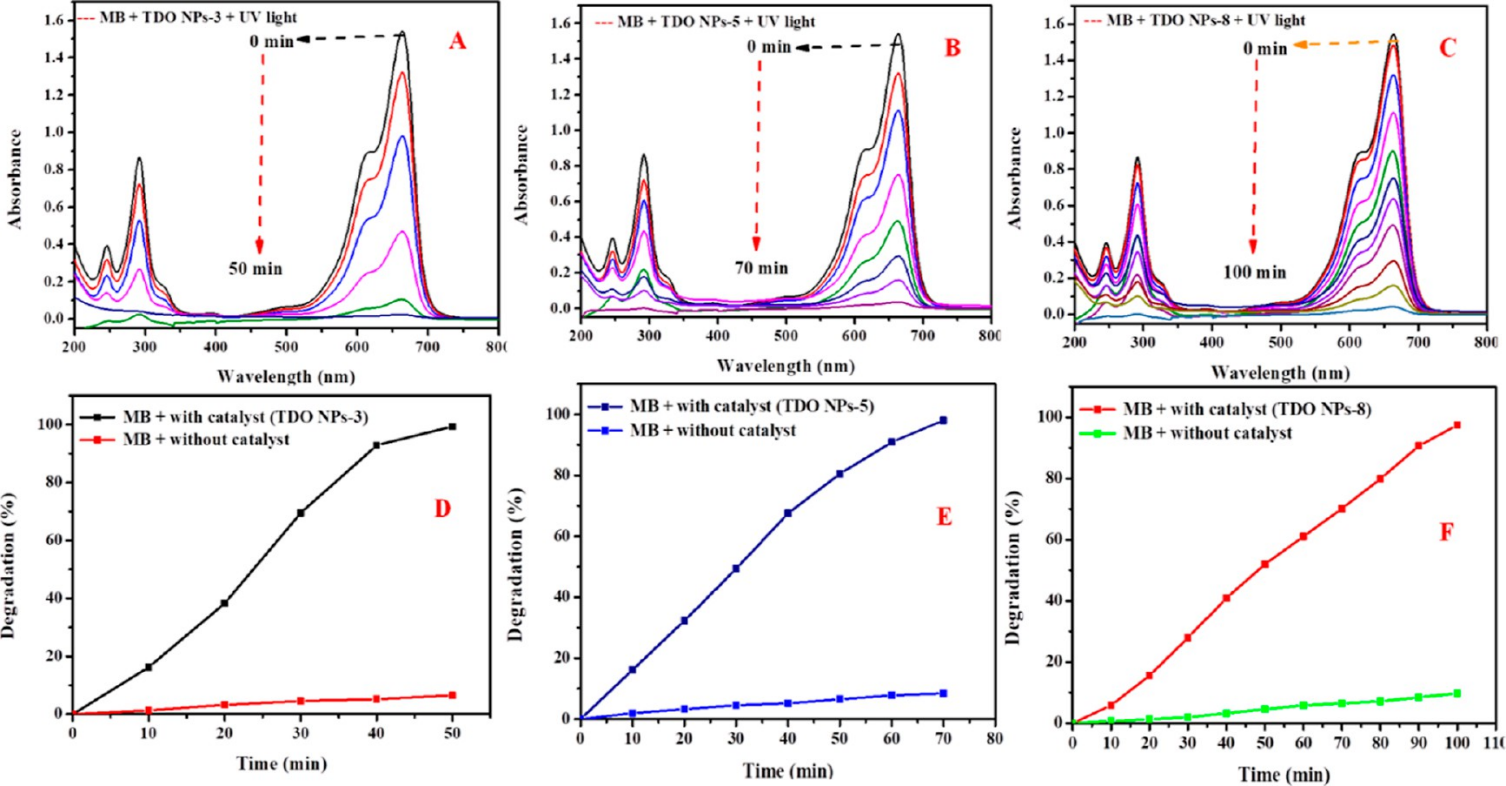
UV–vis absorption spectra of MB dye degradation (A) in the presence of TDO NPs3, (B) in the presence of TDO NPs5, and (C) in the presence of TDO NPs8 under UV irradiation and percentage of MB dye degradation with catalysts and without catalysts in the (D) presence of TDO NPs3, (E) presence of TDO NPs5, and (F) presence of TDO NPs8 under UV irradiation.

The photocatalytic activity of the synthesized TDO NPs was investigated toward degradation of MB dye under UV light irradiation (λ_max_ = 254 nm) inside a Heber multi-photoreactor. Figure 9 presents the UV–visible absorbance spectra recorded from 200 to 800 nm for MB dye under UV irradiation, and after that, sample was collected at regular time intervals. A strong absorption band at 664 nm represents the maximum wavelength of MB dye, which clearly shows a decreasing trend in intensity with time in the presence of TDO NPs3 as a photocatalyst. Diallo et al. (2016) also reported degradation of MB, Congo red, and eosin dye with UV irradiation using TiO_2_ NPs, which supports our present results.^30^ In the present study, MB dye degraded insignificantly (6.49%) without catalysts under identical conditions within 50 min, whereas in the presence of TDO NPs3 photocatalyst, 99.35% degradation was observed under similar conditions. The degradation kinetics was checked as follows eq 3.
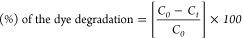
3where *C*_0_ is the initial concentration of the dye and *C*_*t*_ is the concentration of dye at time “*t*” after UV irradiation. The obtained result shows 99.35% MB dye degradation within 50 min under UV irradiation Figure 9A shows the percentage of MB dye degradation with the catalyst (TDO NPs3). In the case of TDO NPs5, 98.05% MB degradation within 70 min was observed, which is represented in Figure 9B. However, in the case of the TDO NPs8 sample, 97.42% MB dye degradation occurred within 100 min, which is shown in Figure 9C.

In the beginning, the MB dye degradation was monitored without a catalyst. The results show that the MB degradation without a catalyst for a sample of TDO NPs3 is 6.49%, under identical conditions within 50 min, which is represented in Figure 9D. In the case of TDO NPs5, 8.44% MB degradation was observed within 70 min, as shown in Figure 9E, whereas in the case of sample TNO NPs8, 9.74% MB degradation occurred, as shown in Figure 9F. Our results are compared with other reported literature studies on degradation of MB and Rh-B dyes and are represented in Table 2.

**Table 2:** Comparison of Photocatalytic Degradation of MB and Rh-B Dyes with Different Nanocatalysts

pollutant dyes	material used	con. NPs/dye	size/shape	time (min)	deg. (%)	references
MB	spindle-like TiO_2_	100.00	50–70 nm/spindle-like	120	62.70	(33)
	SnO_2_/SnO NPs	50.00	14–70 nm/spherical shape	180	90.28	(34)
	CuO microsphere	33.33	31 nm/flower-shaped	360	95.03	(35)
	Sr doped ZnO nano-catalyst	33.33	25–45nm/hexagonal	120	78.50	(36)
	ZnO + rGO nano-catalyst	33.33	15–35 nm/spherical shape	120	92.50	(37)
	Cu/MMT nano-catalyst	31.26	8 nm/spherical shape	120	95.06	(38)
	TDO-nano-catalyst	33.33	5.14 nm/spherical shape	50	99.35	present work
Rh-B	ZnO–SnO_2_ composite	104.38	30 nm/nanofibers	360	49.00	(39)
	CuO-nanowires	83.68	15 nm/wire-like nano	660	95.00	(40)
	Fe_3_O_4_/Zn-/CuWO_4_ nano	83.54	55 nm/spherical shape	210	99.00	(41)
	CuO-nano catalyst	50.00	75 nm/microflakes	300	100	(42)
	SnO_2_ NPs	40.00	7–14 nm/tetragonal	120	95.00	(43)
	ZnO nano-catalyst	15.00	20–30 nm/spherical shape	70	95.66	(44)
	TDO-nano-catalyst	33.33	5.14 nm/spherical shape	60	99.28	present work

### 4.2 PhotoDegradation of Rh-B Dye

**Figure 10:**
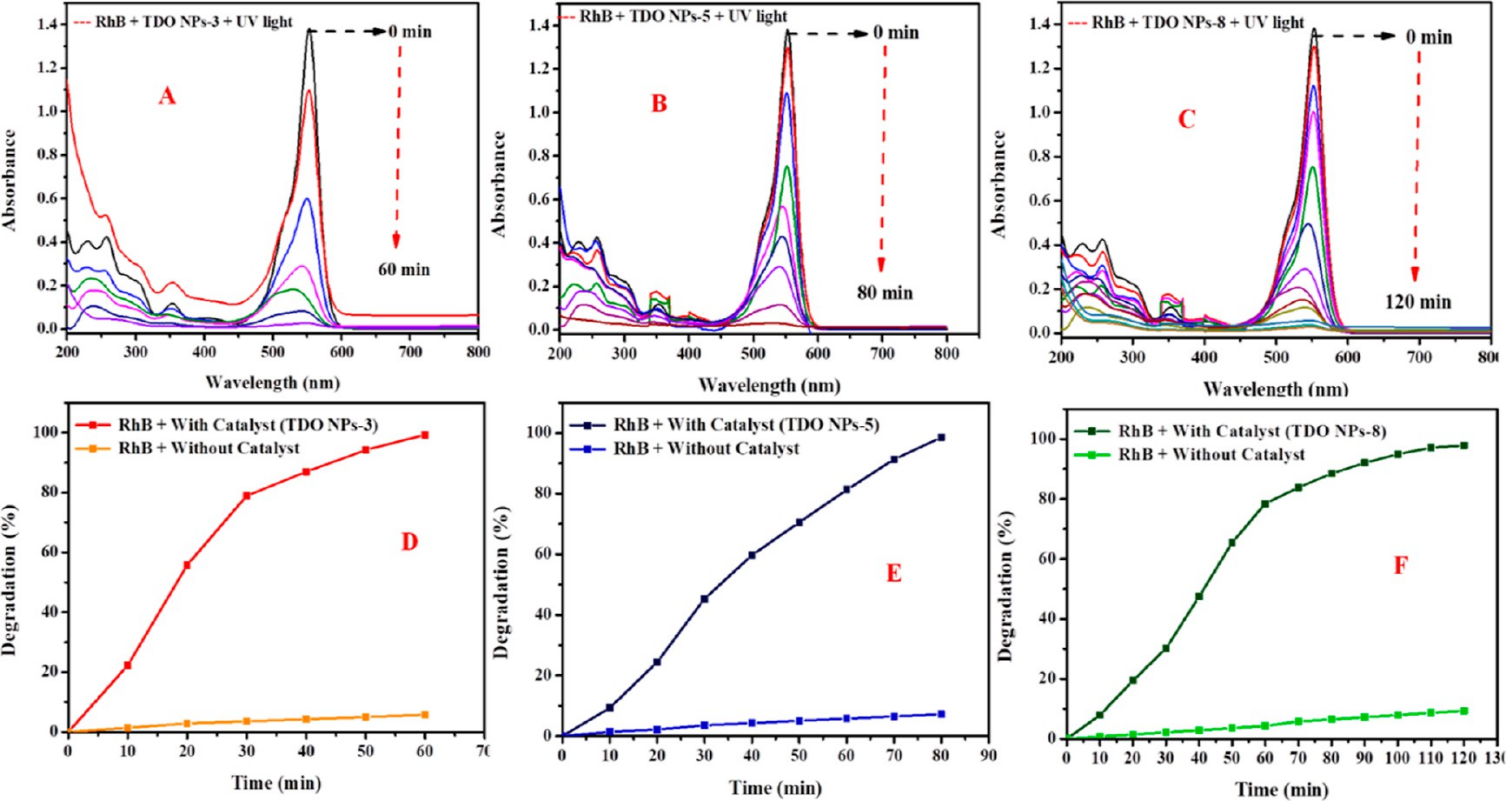
UV–vis absorption spectra of RhB dye degradation (A) in the presence of TDO NPs3, (B) in the presence of TDO NPs5, and (C) in the presence of TDO NPs8 under UV irradiation and percentage of RhB dye degradation with catalysts and without catalysts in the (D) presence of TDO NPs3, (E) presence of TDO NPs5, and (F) presence of TDO NPs8 under UV irradiation.

Figure 10 presents the UV–vis absorbance spectra of Rh-B dye degradation with the TDO NPs3 nanocatalyst under UV-irradiation, where degradation was recorded at a λ_max_ of 552 nm from 200 to 800 nm at regular time intervals. The color intensity of Rh-B significantly changed under UV irradiation. In the presence of a catalyst, Rh-B dye degraded 99.28% within 60 min under UV irradiation. The present results are similar to the degradation of RhB/MB in aqueous solution using nanosized Fe–Cd co-modified ZnO reported by Nea et al. (2018).^31^ The degradation % of Rh-B dye is measured by using eq 3. Figure 10A shows the degradation of Rh-B dye (%) with the catalyst. 99.28% of Rh-B dye degraded within 60 min in the presence of TDO NPs3 as a catalyst. In the case of sample TDO NPs5, 98.56% Rh-B dye degradation was observed, as shown in Figure 10B, whereas in the case of sample TDO NPs8, 97.84% Rh-B dye degradation occurred; this is represented in Figure 10C. In the beginning, the Rh-B dye degradation was monitored without a catalyst. The results show that the Rh-B degradation without a catalyst is 5.75, 7.19, and 9.35% with respective times 60, 80, and 120 min, as shown in Figure 10D–F under identical conditions. The present results are compared with those reported in the literature for the degradation of dyes using nanocatalysts (see Table 2).

### 4.3 Photocatalytic Activity of P25 Nanomaterials for Degradation of MB and Rh-B Dyes

**Figure 11:**
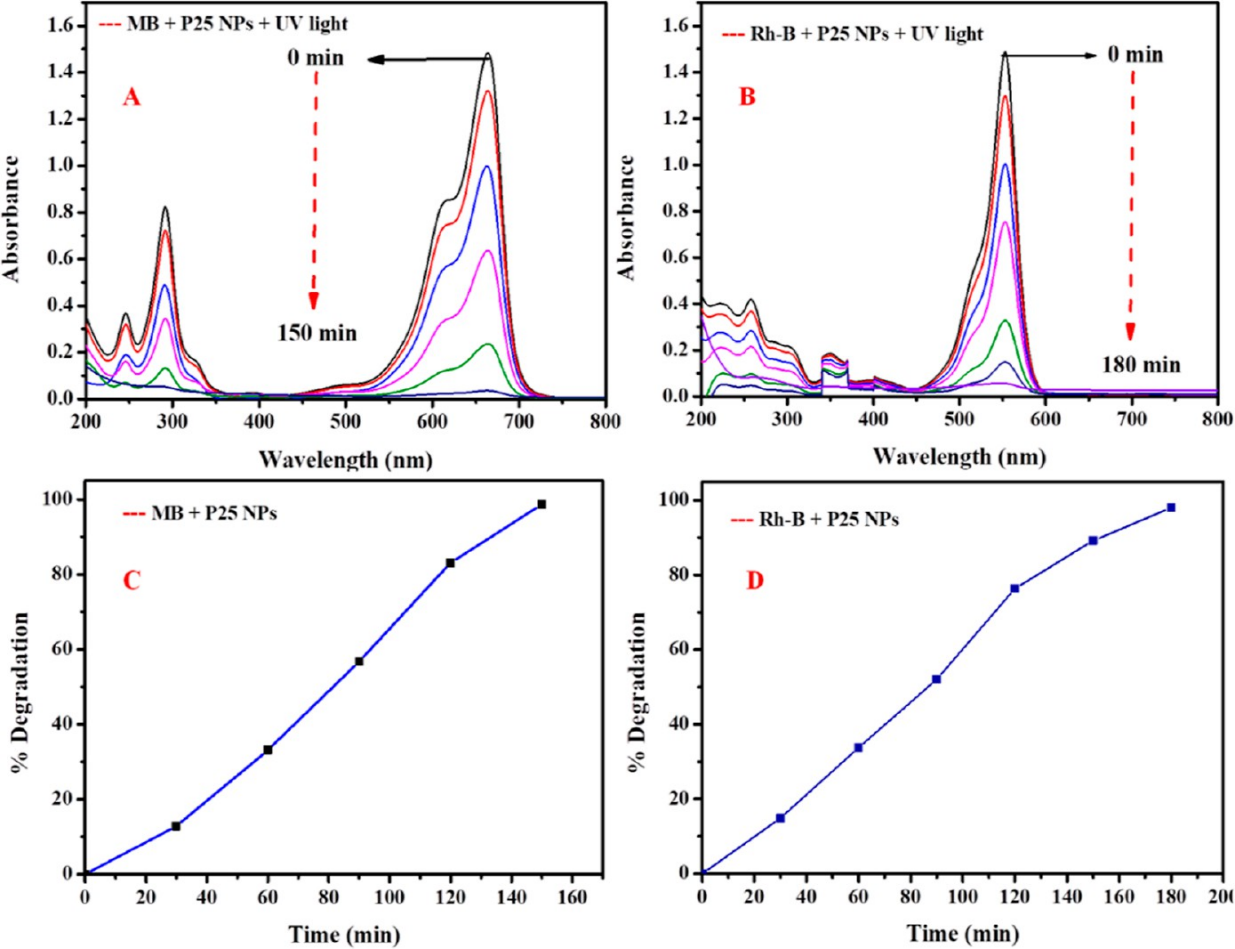
UV–vis absorption spectra of MB dye degradation (A) and Rh-B dye degradation (B) in the presence of commercial P25 NPs and percentage MB dye degradation (C) and percentage Rh-B dye degradation (D) under UV light irradiation.

The photocatalytic activity of the standard commercial photocatalyst (P25 NPs) was tested for degradation of MB/Rh-B. Therefore, the photocatalytic activity of P25 NPs was observed in the presence of UV light (λ_max_ = 365 nm) irradiation for the degradation of MB and Rh-B. Figure 11A,B demonstrates the absorbance spectra of MB and Rh-B dye degradation at regular time intervals (30 min) that were recorded in the range of 200–800 nm. In the presence of a commercial photocatalyst, MB dye degraded 98.64% within 150 min (Figure 11C) and Rh-B dye degraded 89.87% within 180 min (Figure 11D).^32^

The results of this investigation were compared to those published on the degradation of MB and Rh-B dyes by taking into account the ratio of the TDO nanocatalyst dose to dye concentration (mg/mg), NP size and shape, degradation efficiency, and time (min) in each case (Table 2). Likewise, using the SnO/SnO_2_ hybrid photocatalyst, spindle-like TiO_2_, ZnO with the reduced graphene oxide nanocatalyst, and copper NPs on matrix-assisted montmorillonite clays, the nanocatalysis of MB dye degradation has been observed.^33−38^Table 2 summarizes the results reported for ZnO–SnO_2_ composites, CuO-nanowires, Fe_3_O_4_/Zn-/CuWO_4_ nanocatalysts, and ZnO nanocatalysts for the degradation of Rh-B.^39−44^ The degradation duration is obviously dependent on the ratio of the TDO nanocatalyst dose to dye concentration (mg/mg), as well as the size and shape of the NPs (Table 2). Previous research has shown that UV irradiation of MB and Rh-B in the presence of nanophotocatalysts produces a large amount of reactive oxygen species (ROS), which reacts with the dyes to produce non-toxic products such as water molecules, carbon dioxide, and mineral acids, confirming our findings (Table 2). As a result, compared to other findings (Table 2), the synthesized TDO NPs had a higher degrading efficiency due to their smaller particle size and homogeneous spherical shape. As a result of the degradation experiment, we observed that MB degraded in 50 min at a rate of 0.31387 min^–1^, while Rh-B degraded in 60 min at a rate of 0.42525 min^–1^. Under UV irradiation, photocatalytic dye degradation studies were carried out with 20 mg of catalyst and 60 mL of dye solution (10 mg/L). Similarly, a photocatalytic experiment was conducted with various catalyst doses and dye molecule starting concentrations, and the rate constant was calculated (Table 2). As a result, the synthesized TDO NPs demonstrated excellent photocatalytic activity and may be used to degrade poisonous and hazardous organic pollutant dyes.

### 4.4 Effect of the Catalyst Dose on Dye Degradation

**Figure 12:**
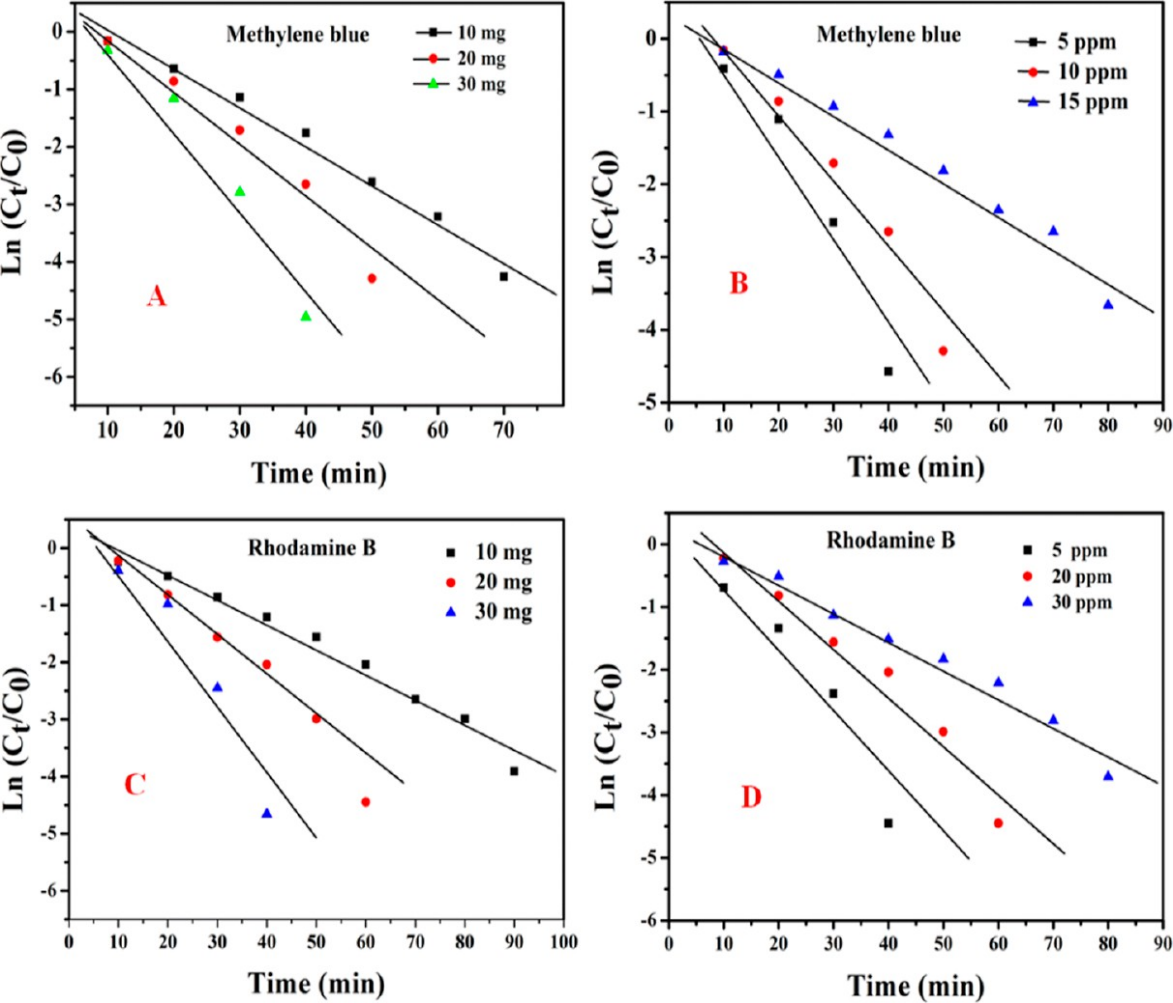
Kinetic plots of MB and Rh-B dye degradation: (A) MB degradation kinetic curves for various catalyst doses (10–30 mg), (B) effect of dye concentration (5–15 mg/L) on the degradation of MB dye, (C) Rh-B degradation kinetic curves for various catalyst doses (10–30 mg), and (D) effect of dye concentration (5–15 mg/L) on the degradation of Rh-B dye.

MB and Rh-B dye degradation was carried out with various sample TDO NPs-3 catalyst doses (10, 20, and 30 mg) at a constant concentration of MB/Rh-B dyes (10 mg/L). The photodegradation efficiency of MB increased from 98.05 to 99.41%, and in a similar way, the Rh-B dye degradation efficiency increased from 97.82 to 99.27% with an increase in the catalyst dose, respectively (Table 3). Photocatalytic dye degradation followed a pseudo-first-order kinetic model as given using the following expression 4.
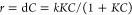
4where “*r*” is the degradation rate of MB/Rh-B, *C* is the concentration of MB/Rh-B solution, *K* is the adsorption coefficient of MB/Rh-B, *t* is the time taken for the degradation, and *k* is the reaction rate constant. For low initial concentrations (*C*_0_ = 10 mg/L in this experiment), this equation can be approximated to a pseudo-first-order model for ZnS:CdS nanocatalyst-mediated photodegradation of MB dye.^45^ As the dose of nanocatalysts increases, the generation of free radicals and the rate constant of the reaction increase, that is, shorter time is required for complete degradation of dyes (Figure 12A,C.

**Table 3:** Effect of Catalyst Doses and Dye Concentration on MB and Rh-B Dye Degradation

pollutant name	con. TDO NPs (mg)/dye (mg)	time (min)	deg. (%)	rate constant (*k*) min^–1^
MB	16.66	80	97.98	0.08546
	33.33	50	99.35	0.14263
	50.00	40	99.42	0.22911
	66.66	40	98.84	0.24252
	33.33	50	99.35	0.14263
	22.22	70	97.63	0.07227
Rh-B	16.66	80	97.09	0.12942
	33.33	60	99.28	0.22605
	50.00	40	98.12	0.35426
	66.66	40	98.44	0.34087
	33.33	60	99.28	0.22605
	22.22	90	96.54	0.15418

### 4.5 Effect of Dye Concentration

The effect of dye concentrations (5, 10, and 15 mg/L) on degradation of MB/Rh-B dye was observed at a constant photocatalyst dose of the TDO NPs3 sample (20 mg). The photodegradation efficiency of MB/Rh-B decreased with increasing dye concentration/doses with respect to time intervals at a constant dose of photocatalysts (Table 3) under UV irradiation, loading with 20 mg of TDO NPs3 sample. Figure 12B,D shows the degradation rate of MB/Rh-B dyes under identical experimental conditions. Chen et al. (2017) proposed a similar photodegradation of dye solution at a constant catalyst dose for ZnO NPs under UV light irradiation.^46^

### 4.6 Plausible Mechanism of Dye Degradation

**Scheme 1:**
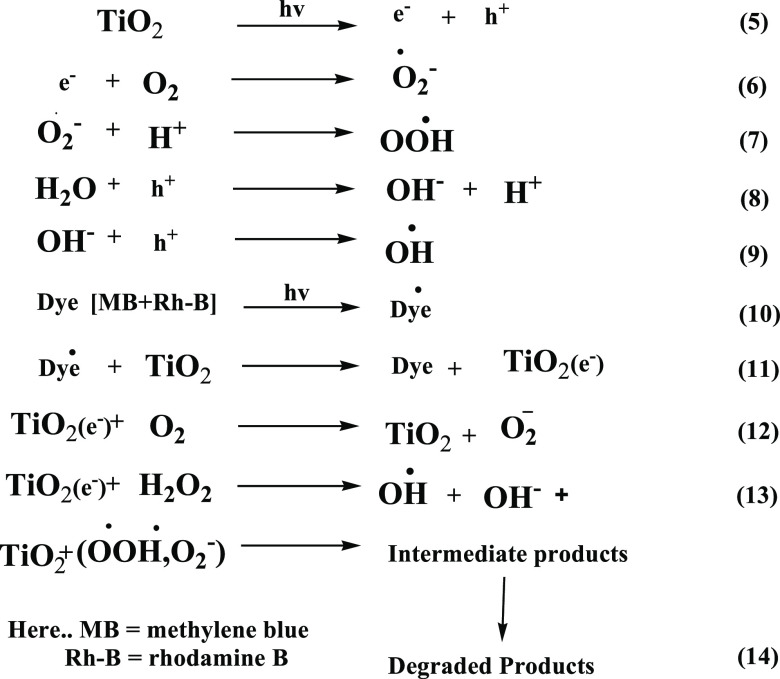
Schematic Representation for the Mechanism of Dye Degradation by Semiconducting TDO NPs Acting as Photocatalysts for the Degradation of MB/Rh-B Dyes

The feasible dramatically represented mechanism of dye degradation was studied for the TDO NPs considering that they are semiconductors and act as a photocatalyst for the degradation of MB/Rh-B dyes (see Scheme 1). Initially, UV irradiation in the presence of TDO NPs generates reactive oxygen species (ROS). Similar situation occurs with ZnO NPs degrading Cong red dye under UV irradiation.^47^

**Figure 13:**
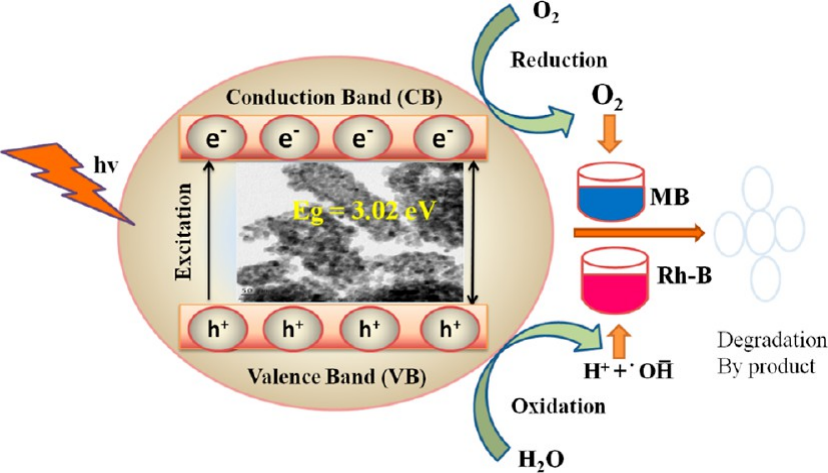
Possible mechanism for MB and Rh-B dye gradation.

It is assumed that TiO_2_ NPs (*E*_g_ = 3.02 eV) excite electrons to the conduction band via valence bands under UV irradiation and generates an electron–hole pair. The conduction band electrons react with oxygen to form superoxide ions (^•^O̅_2_) and the positive holes interact with water molecules to generate hydroxyl radicals (^•^OH) (see eqs 5–9). The generated ROS interacts with dye molecules, leading to degradation to carbon dioxide and water molecules, which are non-toxic. The cleavage of H_2_O_2_ results in hydroxyl radicals (^•^OH), and the photoelectrons reduce the oxygen (O_2_) adsorbed on the photocatalyst surface to produce superoxide radicals (^•^O̅_2_). Eventually, hydroxyl radicals (^•^OH) and superoxide ions (^•^O̅_2_) oxidize/degrade dyes to produce carbon dioxide and water molecules (see eqs 10–14). Zangeneh et al. (2015) reported dye degradation using the radical mechanism under UV irradiation.^48^ A similar mechanism was proposed for the degradation of MO and Rh-B dyes after UV irradiation by using ZnO NPs as catalysts.^49^ The possible mechanism was proposed that is represented in Figure 13. From the results, we concluded that the lower-band gap energy sample (TDO NPs3) shows a quick electron transfer from the valence band to the conduction band, and it shows better results of MB/Rh-B dye degradation within a short period.

## 5 Conclusions

Tinanium dioxide nanoparticles in the anatase phase have been successfully synthesized using an aqueous extract from agro-waste durva grass without the use of toxic chemicals or solvents. The XRD patterns endorse the formation of the crystalline nature of the anatase phase TDO NPs. The average crystallite sizes of the NPs are 6, 8, 26, and 64 nm for the TDO NPs3, TDO NPs5, TDO NPs8, and commercial P25 NP samples, respectively. Further identification by FT-IR spectroscopy shows that the strong stretching vibration at around 420–450 cm^–1^ corresponds to the metal and oxygen bond (Ti-O). HR-TEM images showed spherical shape of all the samples, with average sizes 5.14, 12.54, and 29.61 nm, respectively. The TDO NPs3 sample has a significantly higher zeta potential value (−41.5 eV) compared to that of sample TDO NPs8 (−33.7 eV). Therefore, smaller size of the TDO NPs3 shows the higher stability of NPs. Thermal stability was checked by the TGA technique, which shows that the sample TDO NPs8 exhibits less weight than sample TDO NPs3. The smallest sample (TDO NPs3) has the lowest band gap energy (*E*_g_ ∼ 3.02 eV) under UV irradiation. The TDO NPs3 sample shows good photocatalytic performance for the degradation of MB and Rh-B dyes, with 99.35% degradation within 50 min and 99.28% degradation within 60 min, respectively. Therefore, the TDO NPs3 sample shows better photocatalytic activity than the TDO NPs5, TDO NPs8, and commercial P25 NP samples for the degradation of MB/Rh-B. Eventually, when the amount of catalysts increases from 10 to 30 mg, the rate constant increases and the degradation time decreases. However, when the dye concentration increases from 5 to 15 mg/L, the rate constant gradually decreases and the duration of degradation time increases. As a result, TDO NPs could be used as an effective photocatalyst for the decomposition of toxic dyes in the environment.

## Supporting Information Available

Reusability and stability investigation of TDO NPs and GC-MS analysis of phyto-chemicals (PDF)
