# Supplementary material for: Biogenic Photo-Catalyst TiO2 Nanoparticles for Remediation of Environment Pollutants
Source: ACS Omega. 2022 Jul 20;7(30):26174–89. doi: 10.1021/acsomega.2c01763 (PMC9352162; doi:10.1021/acsomega.2c01763)
Supplement: Supplementary file 1 — ao2c01763_si_001.pdf [file ao2c01763_si_001.pdf]

Supporting Information

Biogenic Photo-catalyst TiO_2 Nanoparticles for Remediation of Environment Pollutants

Boya Palajonnala Narasaiah[1,2#], Pravallika Banoth[1#], Angel Guillermo Bustamante Dominguez[2], Badal Kumar Mandal[3], Challa Kiran Kumar[4], Crispin H.W. Barnes[5], Luis De Los Santos Valladares[2,5,6,]*, Pratap Kollu[1]**

[1]CASEST, School of Physics, University of Hyderabad, Prof. C. R Rao Road, Gachibowli, Hyderabad-500046, Telangana, India

[2]Laboratorio de Cerámicos y Nanomateriales, Facultad de Ciencias Físicas, Universidad Nacional Mayor de San Marcos, Ap. Postal 14-0149, Lima 14, Perú.

[3]Department of Chemistry, School of Advanced Sciences, Vellore Institute of Technology, Vellore-14, Tamil Nadu, India.

[4] Technology Mission Division, Department of Science and Technology, MoS&T, New Delhi, India

[5]Cavendish Laboratory, Department of Physics, University of Cambridge, J.J. Thomson Ave., Cambridge CB3 OHE, United Kingdom.

[6]School of Materials Science and Engineering, Northeastern University, No 11, Lane 3, Wenhua Road, Heping District, Shenyang, Liaoning 110819, People's Republic of China.

E-mail: *Corresponding authors: ld301@cam.ac.uk , pratapk@uohyd.ac.in and badalmandal@vit.ac.in

# Equally contributing first authors

Reusability and Stability of TDO NPs

The reusability of the prepared TDO NPs has been investigated. The TDO NPs were collected by centrifugation after UV-irradiated degradation of MB and Rh-B. They were then washed with double distilled water under the similar experimental conditions. The washed TDO NPs were reused five times in a row for dye degradation. It was discovered that the degradation efficiency of the reused TDO NPs towards degradation of MB and Rh-B dyes was 99.35 % and 99.28 % during the first cycle, but it decreased to 95.18 % and 95.02 % for

MB and Rh-B after the fifth cycle (see **Figure S1A).** As a result of this, we concluded that TDO NPs have a high reusability for the degradation of environmental pollutants dyes. Also, the stability of reused TDO NPs after five cycles was tested using powder XRD, and the results show that there was no change in crystallinity nature of the reused-TDO NPs compared to the fresh ones (see **Figure S1B).**

GC-MS Analysis of Durva grass extract

From GC–MS technique was carried out to screen and noticed phyto-chemicals in agro-waste *durva* grass extract, those are identified phyto-chemicals were responsible for the formation of TDO NPs. Therefore, the two major peaks present at the retention time at 21.32 and 22.94 min in the GC–MS chromatogram. Therefore, retention time at 21.32 min peak indicated aliphatic aster compounds and retention time at 22.94 min peak for aromatic aster compounds and other major peaks also identified i.e. 15.52 min peak may be represented to aromatic heterocyclic compounds, the peak at 17.92 min could assign to boron compounds, the peak at 19.82 min indicated to fatty acids and the noticed peak at 27.68 min represent to be aliphatic aster compounds which are represented in **(Table S1)**. These compounds i.e. phyto-chemicals in durva grass extract were identified by matching with NIST library and could be responsible for the formation of TDO NPs.

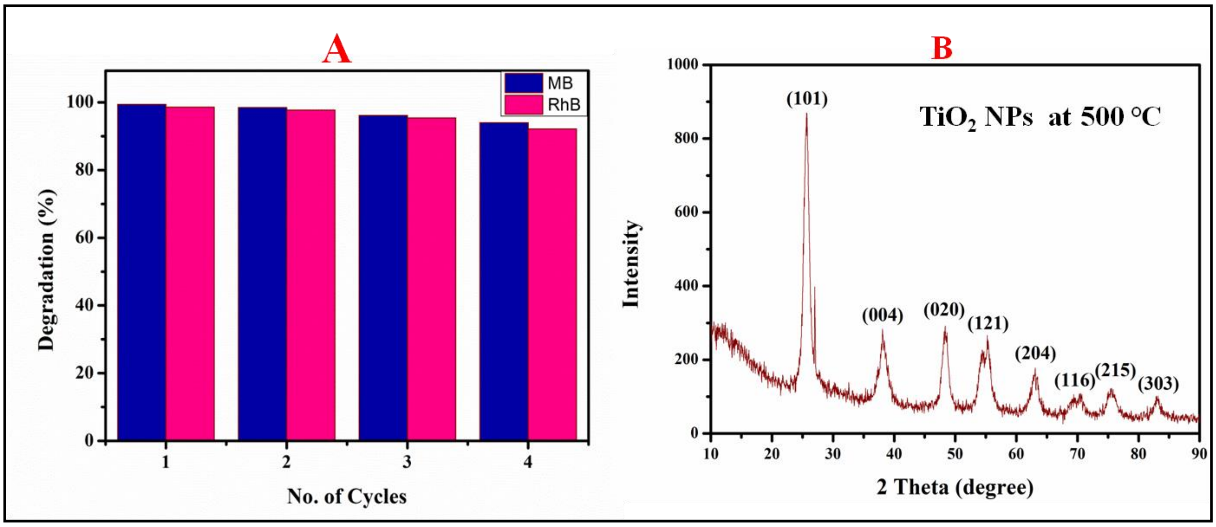

Figure S1. Recyclability checked synthesized TDO NPs-5 for the degradation of MB and Rh-B under identical experimental conditions (A) and check the stability of the TDO NPs-5 sample after degradation of dyes (B)

Table S1. GC-MS analysis of Phyto-chemicals in durva grass aqueous extract

Entry	RT	Structure and Name of the compound	Classification
1	15.52	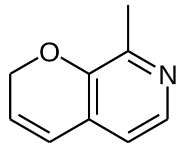 2H-PYRANO[2,3-C]PYRIDINE, 8-METHYL	Aromatic Hetorocyclic compound
2	17.92	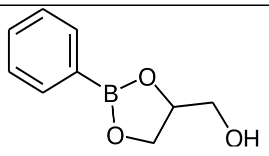 1,3,2-DIOXABOROLANE-4-METHANOL, 2-PHENYL	Boron compounds
3	19.82	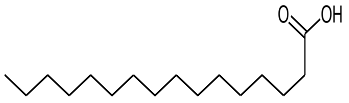 N-HEXADECANOIC ACID	Fatty acid
4	21.32	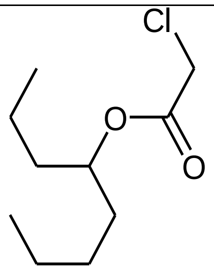 CHLOROACETIC ACID, 4-OCTYL ESTER	Aliphatic aster compound
5	22.94	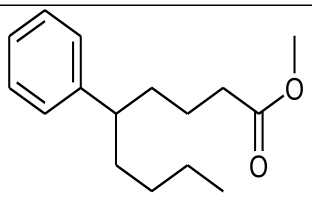 NONANOIC ACID, 5-PHENYL-, METHYL ESTER	Aromatic aster compound

Entry	RT	Structure and Name of the compound	Classification
6	27.68	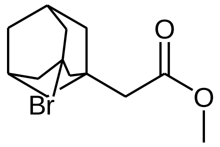<br>METHYL 3-BROMO-1-ADAMANTANEACETATE	Aliphatic aster compound
